# Glioblastoma Stem-Like Cells—Biology and Therapeutic Implications

**DOI:** 10.3390/cancers3022655

**Published:** 2011-06-10

**Authors:** Demirkan B. Gürsel, Benjamin J. Shin, Jan-Karl Burkhardt, Kartik Kesavabhotla, Cody D. Schlaff, John A. Boockvar

**Affiliations:** Laboratory for Translational Brain Tumor and Stem Cell Research, Department of Neurological Surgery, Weill Cornell Brain Tumor Center, Weill Cornell Medical College, New York, NY 10021, USA; E-Mails: bshin84@gmail.com (B.J.S.); jankarl.burkhardt@gmail.com (J.-K.B.); kak2028@med.cornell.edu (K.K.), cschlaff90@gmail.com (C.D.S.)

**Keywords:** glioblastoma multiforme, stem-like cells, brain tumor stem cells, differentiation, culturing, stem-cell hypothesis

## Abstract

The cancer stem-cell hypothesis proposes that malignant tumors are likely to encompass a cellular hierarchy that parallels normal tissue and may be responsible for the maintenance and recurrence of glioblastoma multiforme (GBM) in patients. The purpose of this manuscript is to review methods for optimizing the derivation and culturing of stem-like cells also known as tumor stem cells (TSCs) from patient-derived GBM tissue samples. The hallmarks of TSCs are that they must be able to self-renew and retain tumorigenicity. The isolation, optimization and derivation of TSCs as outlined in this review, will be important in understanding biology and therapeutic applications related to these cells.

## 1. Introduction

There is increasing evidence suggesting that some malignant tumors are likely comprised of a cellular hierarchy that parallels normal tissue. This hierarchy, described by the stem cell hypothesis, consists of stem-like cells, progenitor cells, and terminally differentiated cells. In the case of human gliomas, these tumor stem-like cells (TSCs) are thought to be capable of giving rise to cells that express markers of primary neurons and glial cells such as astrocytes and oligodendrocytes, as well as being able to self-renew [1-4]. Unlike their normal stem cell (NSC) counterparts, TSCs function in a dysregulated manner and are thereby able to repopulate all the cell-types contributing to tumor growth and presumably the inevitable recurrence of glioblastoma multiforme (GBM) [3-5]. However, cancer may or does include non-stem-like cells that still divide and bear certain stem cell characteristics in addition to the subpopulation of stem-like, progenitor and terminally differentiated cells.

While there has been considerable interest in studying TSCs derived from GBM tissue, isolating this sparse cell population with high yield and viability from the greater tumor bulk has been a challenge. These cells have been demonstrated in serum-lacking media containing growth factors, while in suspension they aggregate into spheres. The advantage of this phenomenon is a greater preservation of the native phenotype, genotype and karyotype, which are not preserved in adherent cells because they accumulate aberrations over several passages [5].

While mechanical and enzymatic dissociation has demonstrated moderate success in isolating GBM TSCs, this method entails the introduction of tissue debris in the culture media. Dead cells, resulting from necrosis and mechanical disruption, as well as live red blood cells (RBCs) from vascularized tumors can potentially disrupt sphere formation. Also, live cells will compete for nutrients within the culture media, hence necessitating the removal of these contaminants in order to maximize growth conditions for TSC spheres.

In this review we will discuss our methods to improve the purity and homogeneity of culturing TSCs from patient-derived GBM tissues to further analyze stemness, differentiation, and tumorigenicity *in vitro* and *in vivo* and will compare it with the current available literature.

## 2. Stem Cell Generation

### 2.1. Tissue Preparation and Specimen Procurement

**Figure 1.**
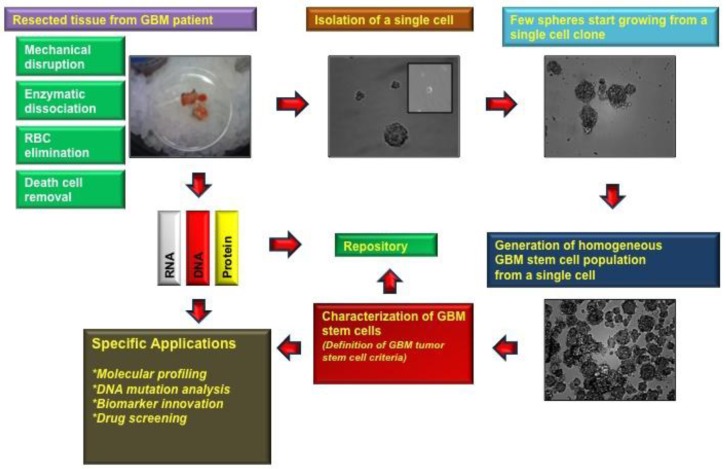
Organizational chart for glioblastoma multiforme (GBM) stem cell and tissue isolation from patient tumor.

After approval by the local institutional review board (IRB) the human tissue sample of interest is stored in normal saline and transported on ice to our brain tumor laboratory after pathological assessment. In a first step the tissue needs to be minced, digested and triturated using Pasteur pipettes several times in order to homogenize the solution (Figure 1) [6-8].

### 2.2. Removal of Red Blood and Dead Cells

RBCs are not the population of interest since they tend to dilute the TSC population and consume the nutrients in the media. To eliminate RBCs, we treat heterogeneous populations with a red cell lysis buffer (Invitrogen, Carlsbad, CA, USA). This solution can lyse anuclear cells, such as erythrocytes, while leaving nucleated cells, such as TSCs in the sample, as previously described by other groups [6,9,10] To be noted, other groups used a Percoll gradient to remove red blood cells and cellular debris [11].

Dead cells are commonly found in the sample due to the expected presence of necrosis in GBM tissue and also due to the mechanical and enzymatic dissociation methods used to isolate the TSCs. In our previous experience with TSCs, these dead cells were found to be a main source for contamination in the stem cell cultures and can potentially disrupt the formation of tumor spheres. In contrast to other groups [7,11] we use a dead cell removal kit (e.g., Miltenyi Biotech), with which it is possible to eliminate the sample of dead cells. Thereafter, cells are ready to be cultured (e.g., approximately 3 × 10^6^ cells plated out per 100 mm dish, and cultures are grown under 5% CO_2_ at 37 °C with a media exchange every 3 days) [8]. Kelly *et al.* for instance, are using the trypan blue staining method to count their viable cells before platting 20,000 cells per microliter without using a dead cell removal kit [6].

## 3. Assessing the Stem Cell Status

**Figure 2.**
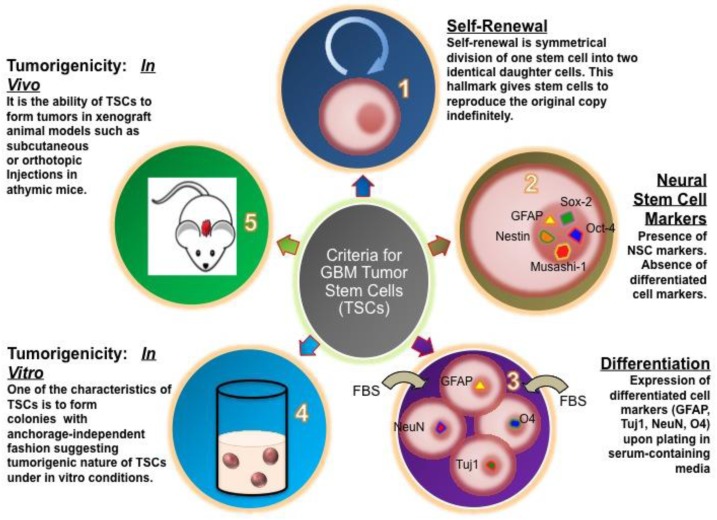
Schematic Presentation of GBM Stem Cell Criteria. The signature characteristics of tumor stem cells (TSCs) are (**1**). self-renewal; (**2**). expression of neural stem cell markers such as Nestin, Sox-2 and Musashi-1; (**3**). differentiation into oligodentrycitic, neuronal and astrocytic populations; (**4**). retaining tumorigenic nature under *in vitro* microenvironment and (**5**). formation of tumors in xenograft transplants in athymic mice.

To verify that cultured glioblastoma cells are stem-like many different methods, described as follows, are available and essential to confirm these characteristics (Figure 2).

### 3.1. Self-renewal/Single Cell Clonal Analysis

Self-renewal is recognized as one of the hallmarks of all stem cells, which enables a single cell to produce two daughter cells as they form spheroids and proliferate indefinitely [12-16]. To generate a homogenous population, a single cell needs to be isolated and plated, for example, in 192 wells per experiment. After a week in culture we usually see in our laboratory that the majority (80%–90%) of the wells contain at least one tumor sphere and continued to expand after approximately 2 weeks. Self-renewal needs to be assayed by serially passaging of spheres in cell culture dishes *in vitro* to justify that sphere-forming cells are able to reform spheres.

### 3.2. Neural Stem Cell Markers

**Figure 3.**
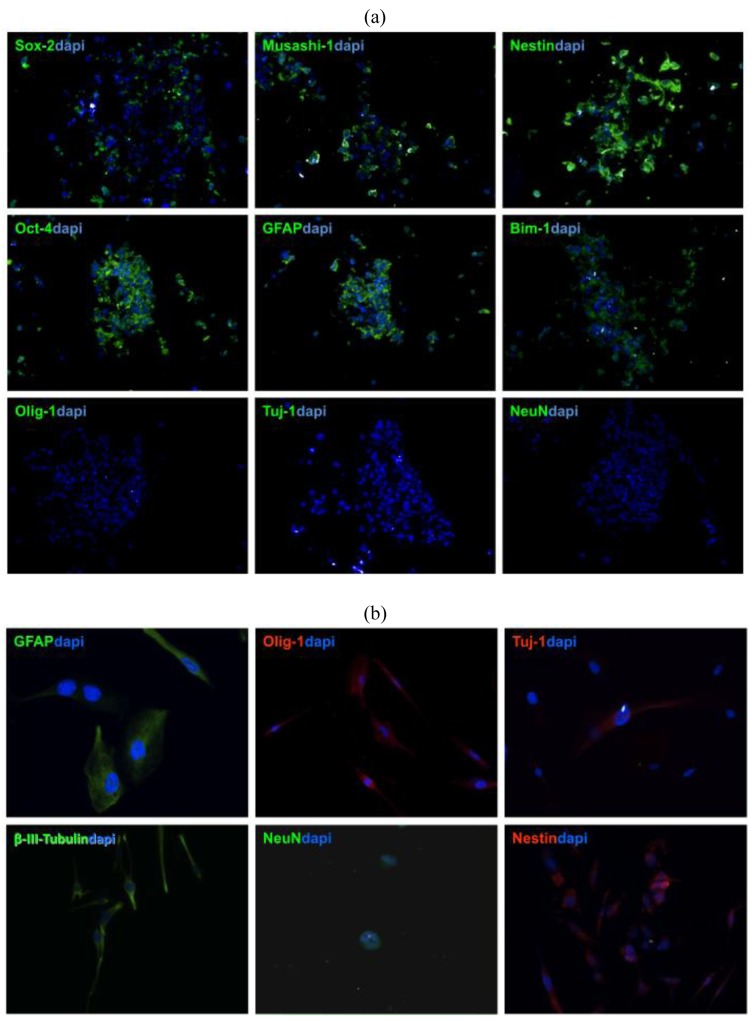
Stemness and Differentiation. Stemness of GBM stem cells is characterized by positive immunoreactivity with Sox-2, Musashi-1, Nestin, GFAP and Bim-1 and absence of expression of Olig-1, Tuj-1 and NeuN (**a**) and induced differentiation of GBM stem cells, positive immunostaining with Olig-1, Tuj-1, NeuN, β-III-Tubulin, GFAP and Nestin (**b**).

To verify that stem cells generated from GBM patient-derived tissue express neural stem cell (NSC) markers, tumor spheres need to be cryosectioned and stained with NSC antibodies [11,13,16]. Patient-derived GBM stem cells show usually strong expression of GFAP, Nestin, Sox-2, Musashi-1, Bmi-1 (Figure 3a), whereas no immuno-reactivity is observed with differentiated cell markers, such as Tuj1, NeuN, which are early and late neuronal markers, respectively, or Olig-1, which is specific for oligodendritic lineages (Figure 3b) [12,13].

### 3.3. Differentiation

The nature of NSCs is that they can differentiate and give rise to neuronal, astrocytic, and oligodendrocytic lineages [14]. To prove this capacity in patient-derived tumor spheres, inducing cell differentiation in media containing FBS needs to be performed. Within a week after exposure to differentiation media, TSCs start to express GFAP, Tuj-1, beta-III-Tubulin, Olig-1 F and later the late neuronal marker NeuN [7,15].

### 3.4. Tumorigenicity of TSCs: *In Vitro vs. In Vivo*

A method to determine whether patient driven TSCs adopt the invasive characteristic of cancer cells, GBM stem cells can be seeded along with normal human NSCs as negative control in soft agar. Normal neurospheres did not grow in soft agar until the third week but began forming colonies toward the end of fourth week. Usually, neural stem cells form small and few colonies anywhere between 4 and 7 weeks after they are implanted into soft agar whereas GBM stem cells start colony formation during the first week [8].

**Figure 4.**
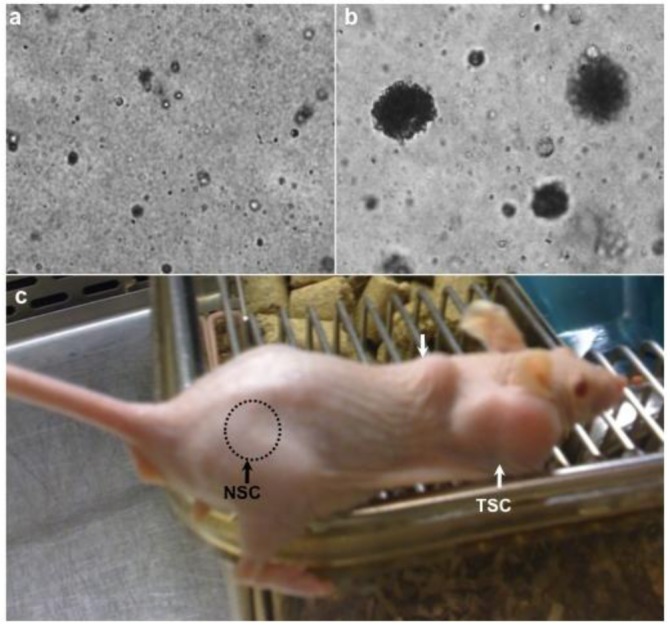
Anchorage-Independent growth and subcutaneous xenografts of GBM stem cells. (**a**) and (**b**) compare normal neurospheres to GBM stem cells, which aggressively develop cell aggregates in 3D experimental system. Tumorigenicity *in vivo* was accomplished when GBM stem cells were injected subcutaneously into the athymic mice (**c**).

To validate if GBM stem cells preserve their tumorigenic character TSCs need to be implanted subcutaneously and intracranially into animals (such as mice or rats), respectively [12,16]. For instance, in our experience, we recorded the tumor volume over 10 weeks and 6 months for the subcutaneous and intracranial injections, respectively (Figure 4). In flank injections, mice receiving 1 × 10^6^ cells per injection developed tumors as early as the fourth week and gradually progressed during the subsequent ten weeks. In the orthotopic injections, mice receiving 100,000 cells per injection showed tumor formation on MRI at 6 months [8].

## 4. Discussion and Perspective

GBM stem-like cells are likely responsible for not only the initiation of GBM but also subsequent recurrences [1-4]. Although isolated populations of GBM tumor spheres is the ideal population for *in vitro* modeling, successful establishment, maintenance, and experimentation on tumor sphere cultures from primary tissue has previously been difficult to perform [2,16,17] In this review, TSCs isolation from human patients with GBM and culture in a specialized stem cell media to promote selective formation of tumor spheres is described.

Tumor stem cells are initially identified by a “neurosphere assay”, which is a set of criteria including the ability to thrive as tumor spheres in a stem cell media containing growth factors but without serum [18,19]. Establishment of a stem-like tumor sphere culture from primary GBM tissue has previously been shown to be problematic, sometimes resulting in a sustainable culture in only half of the processed patient-derived GBM tissue samples [16,20]. In our experience, one of the factors behind the early difficulty of establishing a tumor sphere culture is the presence of other cells and cellular debris. Each culture contains a certain amount of RBCs and more so from resected tissue with increasing vascularity [18]. Live RBCs can compete with the TSC for nutrients in the media and slow proliferation and neurosphere formation. Similarly, increasingly necrotic GBM tumor tissue contains more dead and dying cells or cellular debris. In addition, the mechanical process used to initially dissociate the tissue often destroys TSCs. This cellular debris can be a nidus for contamination and can disrupt sphere formation. Thus, removal of RBCs and other cellular debris from cultures using newer buffers and magnetic bead techniques are paramount for us to obtain pure samples of tumor spheres for our experiments.

Sequential modification and adaptation of our current technique has improved TSC isolation from our GBM patients. The frequency of TSCs isolation from GBM patients has increased from 40% to approximately 90% of patient GBM specimens. By removing RBCs and dying cells, we decrease the quantity of partially or fully differentiated cells [8]. Our findings are different from the findings of Bez *et al.* [21], who showed that neurospheres are made up of a highly heterogeneous population, in which the stem cells at the inner core are less viable.

There is no putative agreement in the scientific community as how to define TSCs; however, there are experimental criteria which are widely recognized as necessary, including a capacity for self-renewal and tumorgenicity [17,18,22]. Other requirements include the presence of stem cell markers and lack of differentiation markers [23,24].

The capacity for self-renewal, thereby implying the ability of clonal proliferation, is suggested by the ability to grow in stem cell media. Self-renewal can be validated through serial passaging of tumor spheres to show that they self-renew [1,25]. To ensure the cells are self-renewing, the stem cells can be separated with each passage from the spheres they originated from and might be suspended as individual cells. For instance, our TSCs are usually able to renew after a single passage, a third passage, and a fifth passage [1,23]. The disadvantage of this assay, however, is that stem cells are not the only ones that are capable of forming neurospheres. The tumor sphere population is heterogeneous and committed progenitor cells can form neurospheres. As a result, complementary assays are required to prove the stem cell nature of these cells.

The antigenic profile of NSCs has been well established, but there is still no single definitive immunophenotype that can be attributed to TSCs. TSCs should stain not only for relevant stem cell markers, but also stain negative for differentiation markers [23,26]. TSCs may stain positive for GFAP, an astrocytic marker that has been shown to stain strongly positive in NSCs, as well as Nestin, Sox2, and Musashi-1, which are all conventional NSC markers [26-28]. TSCs are usually negative for the relevant differentiation markers including, Tuj-1 and NeuN, which are early and late neuronal markers respectively, as well as O4, an oligodendritic marker. The expression of GFAP in GBM TSCs is not well established. While there is some consensus that neural stem cells may express GFAP [5], Lee *et al.* put forth the idea that GBM TSC spheres only regain GFAP expression after differentiation (not reflective of the primary tumor cells). However, Gunther *et al.* and Bleau *et al.* more recently have provided evidence that undifferentiated GBM tumor spheres express normal brain stem cell markers, including GFAP [1,29]. Prestegarden *et al.* showed that GFAP positive cells formed tumors with no difference in survival rates [30]. Our experience with TSC in this regard has been in concordance with the notion that GBM TSCs strongly express GFAP before differentiation. This protein marker profile further confirms that the tumor spheres generated in our experiments were TSCs [8].

The presence of CD133 or Prominin-1 antigen, a transmembrane protein with an unknown function, has been described as a definitive antigen in identifying human GBM derived TSCs. However, recent evidence has shown that the population of cells that are CD133 negative are equally tumorigenic when xenografted into immunodeficient mice [19,31]. CD133 antigen should be seen as a prognostic marker, and the presence of this antigen could indicate resistance to chemotherapy and ionizing radiation due to increased activity at the DNA damage checkpoint [1,22,23,32-34].

TSCs may give rise to cells deprived of stem properties and presenting some common markers with oligodendrocytes or astrocytes [23,25,35-37]. When cultured in media containing serum, tumor spheres lose their spherical morphology while adopting various adherent morphologies and staining positive for a number of differentiation markers. In our experience, TSCs usually exhibited some Tuj-1 positively within the first days, indicating early neuronal development. However, in this early stage TSCs did not stain for NeuN, a late neuronal marker. Cells also stain positive for O4 in this early stage, showing that the TSCs also differentiated into the oligodendritic subtype. Filamentous GFAP staining is usually observed in our TSCs, which is consistent with the fact that GBMs are mostly comprised of differentiated astrocytes [8]. *In vitro* multipotency of TSCs is limited only to the mature cell types in the original tumor [22,34].

The greater tumorigenicity of TSCs theoretically results from altered genetics, resulting in increased aberrant activation of signal transduction pathways or decreased disruption of cell cycle arrest [38]. Understanding these mutated genes in TSCs would expand our knowledge of the mechanisms of gliomagenesis and offer direction into possible therapies targeting TSCs [39]. For example, gene replacement therapy could potentially be an invaluable tool for *in vivo* treatment of GBMs. One of the main limitations, however, of using gene replacement therapy for the treatment of brain tumors is the difficulty of gene delivery to the relevant cells within the tumor. However, if for instance a stable gene that induces apoptosis or suppresses pro-oncogene were introduced into the TSCs of a GBM, it may prevent TSCs from recapitulating the tumor after treatment. If TSCs were effectively treated, even a residual tumor might eventually degenerate [4]. This theory, however, requires a way of specifically targeting resident TSCs within GBM.

## 5. Methodology Review

### Preparation of tissue

The tissue was digested in papain (Worthington, Lakewood, NJ, USA) and DNase I (Sigma-Aldrich Co., St. Louis, MO, USA) cocktail for one hour at 37 °C. The pellet was resuspended in DMEM/F12 containing EGF (20 ng/mL), bFGF (20 ng/mL), B27 (Invitrogen, Carlsbad, CA, USA) and antibiotic/antimycotic (Invitrogen, Carlsbad, CA, USA). The mixture was filtered through a 70 μm cell strainer, enzymatic reaction was stopped by adding 5% FBS to the solution. To eliminate RBCs, the pellet was resuspended for 10 min in 10 mL red cell lysis buffer (Invitrogen, Carlsbad, CA, USA) and for removal of dead cells, dead cell removal kit, was applied according to manufacturer's suggestions (Miltenyi, Auburn, CA, USA).

### Self-renewal

Tumor spheres were mechanically dissociated then plated out as single cells. All the cells were refreshed with medium every 3 days.

### Cryosectioning Embedded Cells and Immunocytochemistry

TSCs were washed in PBS, fixed in 4% paraformaldehyde for 5 min and a drop of cells was embedded at the center of a block of Tissue Tek—Optimal Cutting Temperature embedding media (Sakura, Torrance, CA, USA). Tumor spheres were sectioned at 7-μm slices, were blocked in 1 × PBS with 0.1% Tween (Invitrogen, Carlsbad, CA, USA) and 5% normal goat serum (for cytosolic/surface antigens)] for 90 min and incubated overnight at 4 °C in neural stem cell antibodies; Musashi-1, Sox-2, Nestin, GFAP, Bmi 1. For transcription antigens, the spheres were treated with 0.1% Triton X-100 for 1 hour at room temperature. All samples were incubated with primary antibodies overnight at 4 °C followed by secondary antibody incubation the next day. Prepared samples were examined by using fluorescence microscopy or a monochrome digital camera.

TSC differentiation was established when 10% FBS was added to the media in the absence of growth factors. At different time points, TSCs cells were trypsinized, seeded on cover slips, fixed in 4% PFA and incubated with differentiation markers such as GFAP, Tuj-1, NeuN, O_4_, Olig-1, b-III-Tubulin.

### Colony Formation in Soft Agar

TSCs at 105 density were mixed with 0.3% agar suspension with DMEM/F-12 containing EGF (20 ng/mL), bFGF (20 ng/mL), B27, and antibiotic/antimycotic per 60 mm dish. Cells were re-fed twice per week with a top agar suspension containing fresh growth factors. Colonies were counted from 7 independent fields by two investigators, and photographed at the end of the second and third week.

### Tumorigenicity of TSCs

For subcutaneous injections, TSCs and NSCs were deposited in 100-μL PBS containing 25 × 10^6^, 5 × 10^5^ or 1 × 10^6^ cells per injection and each mouse received two injections, one with TSCs and the other with NSCs isolated from the human hippocampus or neocortex samples serving as a negative control.

## 6. Conclusions

We review the process of harvesting and isolating stem-like cells from human glioblastoma tissue. Our techniques have proven to be an efficient way to increase the yield of stem-like cells. These cells are not only important for further *in vitro* and *in vivo* understanding of glioblastoma tumor biology but also for designing therapeutic strategies.
